# Novel Nanoparticulate and Ionic Titanium Antigens for Hypersensitivity Testing

**DOI:** 10.3390/ijms19041101

**Published:** 2018-04-06

**Authors:** Paul Johan Høl, Einar K. Kristoffersen, Nils Roar Gjerdet, Amanda S. Pellowe

**Affiliations:** 1 Department of Clinical Medicine, University of Bergen, N-5021 Bergen, Norway; 2 Department of Orthopaedic Surgery, Haukeland University Hospital, Jonas Lies vei 87, N-5021 Bergen, Norway; 3 Department of Clinical Science, University of Bergen, N-5021 Bergen, Norway; einar.kristoffersen@uib.no; 4 Department of Immunology and Transfusion Medicine, Haukeland University Hospital, N-5021 Bergen, Norway; 5 Department of Clinical Dentistry, University of Bergen, N-5009 Bergen, Norway; gjerdet@uib.no; 6 School of Engineering and Applied Sciences, Yale University, New Haven, CT 06511, USA; Amanda.pellowe@yale.edu

**Keywords:** allergy, hypersensitivity, titanium, cytokine, lymphoproliferation, flow cytometry, implant, multiplex bead assay, adverse effects of nanomaterials on immune system

## Abstract

Titanium is used in a wide variety of materials ranging from medical devices to materials used in everyday life. Adverse biological reactions that could occur in patients, consumers, and workers should be monitored and prevented. There is a lack of available agents to test and predict titanium-related hypersensitivity. The aim of this study was to develop two bioavailable titanium substances in ionic and nanoparticulate form to serve as antigens for hypersensitivity testing in vitro. Peripheral blood mononuclear cells from 20 test subjects were stimulated with the antigens and secretion of monocytic and lymphatic cytokines and chemokines were measured by a multiplex bead assay. Lymphocyte stimulation indices were also determined in a subset of test subjects by measuring CD69 and HLA-DR expression by flow cytometry. Cytokine profiling revealed that both antigens increased production of typical monocyte and macrophage secreted cytokines after 24 h, with significant increases in IL-1β, IL-7, IL-10, IL-12, IL-2R, IL-6, GM-CSF, TNF-α, IL-1RA, MIP-1α, MIP-1β, IFN-α, and IL-15. Lymphatic cytokines and chemokines were not significantly induced by activation. After seven days of stimulation, ionic-Ti (2.5 μg/mL) caused proliferation (stimulation index > 2) of CD4+ cells and CD8+ cells in all persons tested (*N* = 6), while titanium dioxide nanoparticles (50 μg/mL) only caused significant proliferation of CD4+ cells. Our preliminary results show that the experimental titanium antigens, especially the ionic form, induce a general inflammatory response in vitro. A relevant cohort of test subjects is required to further elucidate their potential for predictive hypersensitivity testing.

## 1. Introduction

Titanium and its oxides are utilized broadly for the creation of materials, and it is estimated that 1.45 million metric tons were produced in the United States in 2007 [1]. The unique material properties of titanium dioxide (TiO_2_), such as its high strength and ease of machinability, explain its extensive use for creation of materials including paint, cosmetics, sunscreen, toothpaste, plastics, paper, textiles, food packaging, and medical devices [2,3]. Therefore, exposure to titanium nanoparticles and titanium materials is broad and affects consumers, patients, and people with occupational exposure [4,5,6]. Titanium materials were historically considered bio-inert, since metallic titanium is highly resistant to corrosion. However, in the last two decades the risks associated with titanium exposure have been questioned in response to accumulating evidence of occupational and medicinal risks. In 2006, the International Agency for Research on Cancer classified TiO_2_ as possibly carcinogenic to humans [7].

With the rapid development of nanotechnology, engineered TiO_2_ nanoparticles (NPs) have become increasingly common, presenting additional exposure risks. NPs are defined as having particle diameters less than 100 nm. Due to their small diameters, NPs are respirable and have a large surface area to mass ratio, imparting an increased chemical reactivity. The National Institute for Occupational Safety and Health has concluded that surface area is a critical metric for assessing occupational respiratory exposure to TiO_2_, although the extent that these properties specifically contribute to exposure risks has been debated [1,8]. Alternate NP exposure routes include topical (skin), gastro-intestinal, and through wear and biocorrosion of medical implants. Once inside the body, opsonization of metallic particles initiates monocyte/macrophage activation and subsequent release of pro-inflammatory cytokines to recruit additional immune cells and promote immunological activity, such as phagocytosis [9,10,11]. Both lymphocyte accumulation and macrophage infiltration have been reported in the vicinity of titanium implants [12,13]. Inhalation is the most common occupational exposure to TiO_2_, and assessment of bronchoalveolar lavage (BAL) fluid after intratracheal administration of TiO_2_ nanoparticles in vivo induce recruitment of inflammatory cells, including neutrophils, which are also known to phagocytose foreign pathogens and secrete granular contents that can induce off target tissue damage [14]. Pulmonary inflammation, fibrosis, epithelial hyperplasia, and tumorigenesis have all been reported in relation to dose and duration dependent models of in vivo TiO_2_ exposure [8]. Thus, NPs may be significant contributors to immune responses, and interactions between NPs and immune cells represent an emerging category of safety concern [15,16].

In addition to TiO_2_ NPs, titanium ions are also known to cause immune responses because of their ability to bind to proteins, such as albumin or transferrin [17,18,19], creating an immunogenic adduct capable of stimulating the immune system [20,21]. It is likely that ionic titanium released from implants is stabilized by biomolecules such as citrate, which is one of the most important low-molecular-mass metal chelators in cellular fluids, having a concentration of approximately 0.1 mM in human plasma [22]. The coordination complexes of Ti(IV) citrate are relatively stable to hydrolysis in aqueous solutions near neutral pH. However, it has been shown that proteins like transferrin and albumin can exchange with the citrate ligand to create a bioavailable metalloprotein [23] that could serve as an antigen in immunological reactions. Current methods for testing immune sensitivity and the specific mechanisms of immune response to titanium NPs and ions are limited, partly due to the lack of in vitro methods for synthesizing and evaluating human immunity. The current method for predicting metal allergy is epicutaneous patch testing, although these tests can give false positive or false negative results. Further, patch testing is rarely predictive of immunity from internal exposure due to the low solubility of titanium salts and metals, thus reducing the ability of the metal to penetrate the skin [11,24,25,26]. It has been shown that stable complexes of TiO_2_-nanoparticles bound to proteins can be produced in vitro under physiological conditions, and these are candidates for use as antigens for in vitro assessment of human immunity [27,28]. Additionally, in vitro reactivity to more bioavailable titanium antigens may provide an alternative method for detecting unwanted immune responses or serve as a novel method to study immunogenic cascades induced by TiO_2_ exposure.

Flow cytometry is a proven method for determining the lymphocyte stimulation index as a measure of hypersensitivity to metals [29]. Additionally, the CD69 (cluster of differentiation 69) surface antigen is one of the earliest markers of hypersensitivity and can be detected as early as 24 h after stimulation, [30,31] and class II tissue type antigens like HLA-DR (human leukocyte antigen—antigen D related) are often expressed on activated antigen presenting cells, including macrophage infiltrating cells [10]. Thus, lymphocyte stimulation indices and expression levels of CD69 and HLA-DR serve as metrics for quantitative analysis of cellular hypersensitivity. Cytokine expression levels can also be used as biomarkers of immunomodulatory properties. Many studies have shown that proinflammatory cytokines such as IL-1β (interleukin 1beta), TNF-α (tumor necrosis factor-alpha), and GM-CSF (granulocyte-macrophage colony-stimulating factor) are co-upregulated after hapten or particle stimulation [32,33,34,35,36]. Other cytokines—including IL-6 (interleukin 6), IFN-γ (interferon gamma), IL-12 (interleukin 12), and IL-15 (interleukin 15)—are also linked with contact sensitivity or particle stimulation [37,38,39]. These studies have been helpful in determining responses from general metal sensitivity, but there is limited literature that specifically addresses human response to titanium [40,41]. Most studies that include titanium focus on particulate titanium and exclude ionic titanium completely. Due to the lack of consensus on cytokine responses to metals and the limited knowledge available about hypersensitivity to titanium, a high throughput method would be advantageous to measure a wide range of monocytic and lymphatic cytokines and chemokines in response to novel titanium antigens in vitro.

In this study, we aimed to develop two bioavailable titanium substances in ionic and nanoparticulate forms and test their potential as antigens for hypersensitivity testing in vitro. In a small pilot, we tested the responses induced by these novel titanium antigens in 20 test subjects by measuring lymphocyte stimulation indices, CD69 and HLA-DR expression, and a wide range (multiplex) of both monocytic and lymphatic cytokines and chemokines in vitro.

## 2. Results

### 2.1. Endotoxin Levels

All test substances were tested for endotoxin levels and were found to be acceptable and below 1 EU/mL. Human serum albumin (HSA) was found to be the reagent that contributed to the endotoxin level in the Ti-antigens, but HSA alone (negative control in *N* = 3 experiments) did not stimulate cytokine release.

### 2.2. Lymphocyte Proliferation Responses to Titanium Antigens

**Figure 1:**
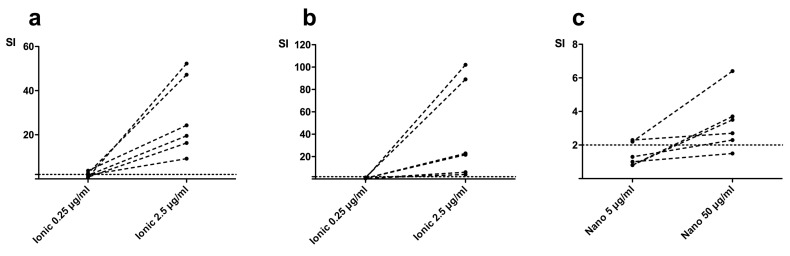
Lymphocyte proliferation (stimulation index (SI) = % of stimulated CFSE positive cells relative to the mean % of unstimulated control cells) of CD4+ cells and CD8+ cells after stimulation with ionic-Ti (**a** (CD4+), **b** (CD8+)) and nano-TiO_2_ (**c** (CD4+)). (*N* = 6).

PBMCs from all test subjects showed proliferative responses to positive controls PMA/I (phorbol-myristate-acetate and ionomycin), PHA (phytohemagglutinin), and PPD (purified protein derivative) (stimulation index (SI) >2) after seven days of stimulation. After seven days of stimulation with the low concentration of ionic-Ti, two test subjects (2/6) showed significant proliferation of CD4+ cells (Figure 1a). There were no subjects with significant proliferation of CD8+ cells after stimulation with the low concentration of ionic-Ti. The high concentration of ionic-Ti caused significant proliferation in all test subjects for both CD4+ cells (Figure 1a) and CD8+ cells after seven days of stimulation (Figure 1b). Stimulation with the low concentration of nano-TiO_2_ for seven days caused significant proliferation of CD4+ cells in two test subjects (2/6) (Figure 1c) but no test subjects had significant proliferation of CD8+ cells. Stimulation with the high concentration of nano-TiO_2_ for seven days caused significant CD4+ proliferation for all but one patient (5/6) (Figure 1c), and only induced significant CD8+ proliferation for two test subjects (2/6).

### 2.3. Activation Marker Expression

**Figure 2:**
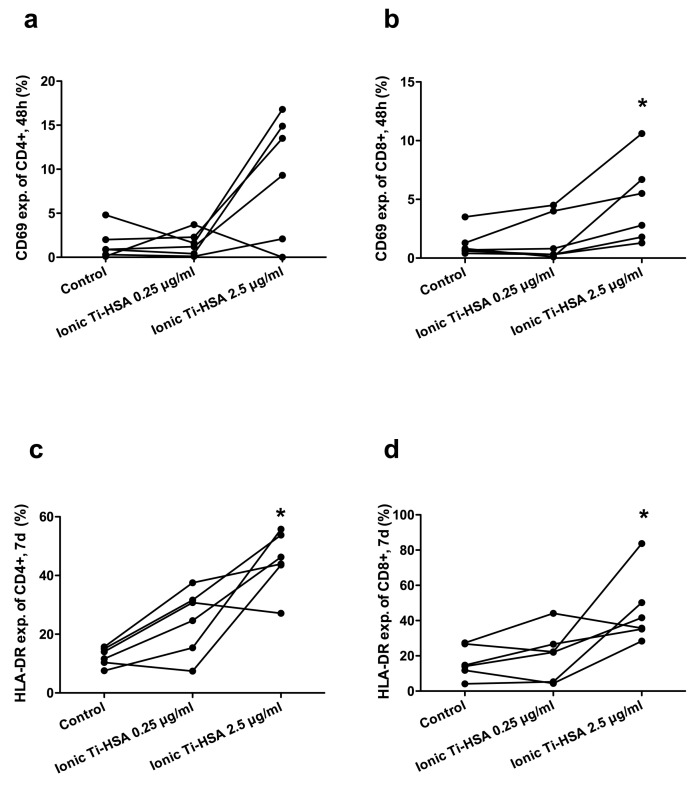
Expression of CD69 after 48 h (**a**,**b**) and HLA-DR after seven days (**c**,**d**) on CD4+ cells (**a**,**c**) and CD8+ cells (**b**,**d**) after stimulation with ionic-Ti (*N* = 6). * Wilcoxon matched pair, *p* < 0.05.

After 48 h of activation, the lowest concentrations of ionic titanium antigens failed to induce CD69 expression on either CD4+ cells or CD8+ cells (Figure 2a,b). With higher concentrations of ionic titanium antigens, CD69 expression was increased in both cell types, however the increase was only statistically significant in CD8+ cells (Figure 2b). HLA-DR expression was significantly increased after stimulation with ionic-Ti. After seven days of stimulation with the high concentration of ionic-Ti, CD4+ (Figure 2c) and CD8+ cells (Figure 2d) had an increase in HLA-DR expression when compared to controls. There was no significant difference in CD69 expression or HLA-DR expression for CD4+ or CD8+ cells after stimulation with nano-TiO_2_ for seven days.

### 2.4. Cytokine Release in Response to Titanium Antigens

PBMCs from all test subjects had increased expression of most cytokines in response to positive controls PMA/I (except for Eotaxin, MCP-1 (Monocyte Chemoattractant Protein-1), IP-10 (Interferon Gamma-Induced Protein-10), IL-8, IFN-γ and IL-6) and PHA (except for Eotaxin, IL-17, MCP-1, IFN-γ) compared to unstimulated cells.

**Figure 3:**
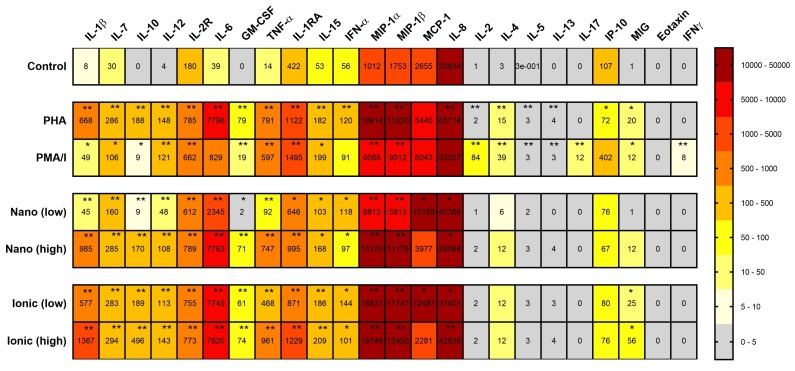
Statistical significance and median concentration (pg/mL) levels for cytokines and chemokines after PBMC stimulation in 24 h with low and high concentrations of nano-TiO_2_, ionic-Ti and positive controls (PHA and PMA) versus unstimulated cells. * *p* < 0.05, ** *p* < 0.001 compared to control using Wilcoxon signed rank test. Abbreviations: interleukin (IL), receptor (R), granulocyte-macrophage colony-stimulating factor (GM-CSF), tumor necrosis factor (TNF), interferon (IFN), macrophage inflammatory protein (MIP), monocyte chemoattractant protein (MCP), interferon gamma induced protein-10 (IP-10), monocyte induced by gamma interferon (MIG).

**Figure 4:**
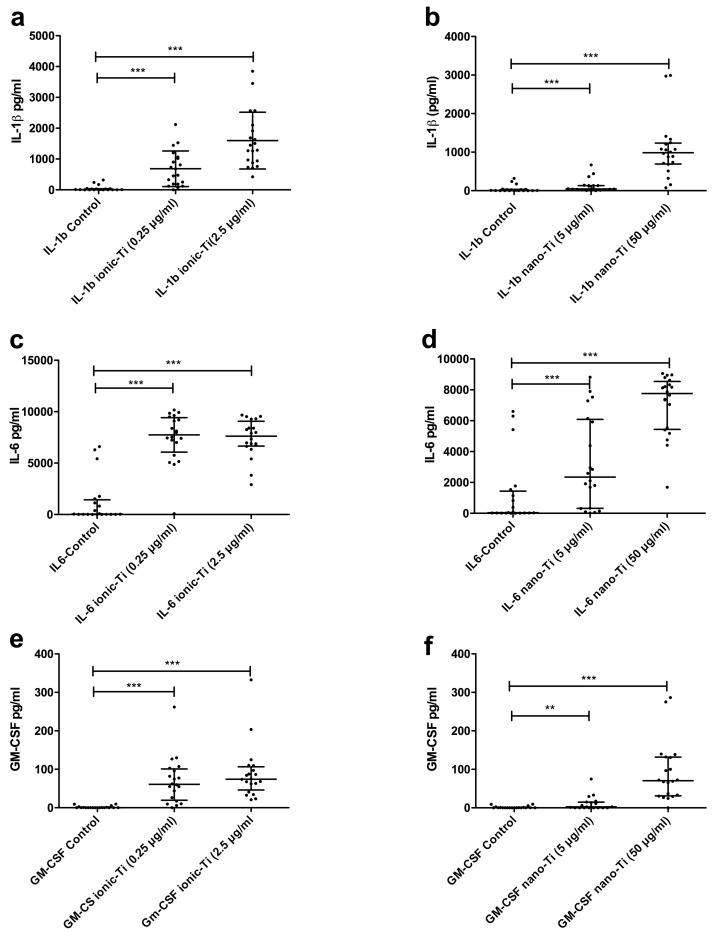
Expression levels of IL-1β (**a**,**b**), IL-6 (**c**,**d**), and GM-CSF (**e**,**f**) were increased after stimulation with either nano-Ti or ionic-Ti for 24 h. Graphs show median and interquartile range (*N* = 20). *** *p* < 0.0001, ** *p* < 0.01, * *p* < 0.05.

All test subjects had increased cytokine expression for a variety of cytokines after in vitro exposure to nano-TiO_2_ and ionic-Ti for 24 h. A summary of all cytokine responses versus unstimulated controls is shown in Figure 3. Out of the 24 cytokines measured, 16 cytokines were significantly increased after stimulation with the nano-TiO_2_ and ionic-Ti: IL-1β (Figure 4a,b), IL-7, IL-10, IL-12, IL-2R (interleukin-2 receptor), IL-6 (Figure 4c,d), GM-CSF (Figure 4e,f), TNF-α, IL-1RA, MIP-1α (macrophage inflammatory protein), MIP-1β, IL-15, IFN-γ, MCP-1, IL-8 and MIG (monokine induced by gamma interferon) (only ionic-Ti). 

## 3. Discussion

Exposure to both nanoparticulate TiO_2_ and ionic titanium poses risks, and a better understanding of the adverse biological reactions from these materials will enhance both monitoring and prevention of related health risks associated with the exposure. The exposure can be endogenous from surgically implanted devices, such as hip and knee prostheses, or exogenous, such as through skin contact or inhalation. Previous studies have shown elevated lymphocyte stimulation indices in patients with problematic titanium implants, and occupational pulmonary exposure to TiO_2_ via inhalation is commonly associated with adverse pulmonary outcomes including fibrosis and cancer. Inspired by these findings, we sought to investigate whether or not our newly developed Ti-antigens with improved bioavailability and stability could be used in modern in vitro assays to reveal information about the immunological potential of titanium substances [11].

### 3.1. Antigens

Titanium has historically been considered bio-inert in both its pure form and as an alloy due to the passivating titanium dioxide layer that spontaneously forms after exposure to oxygen. While this remains true for bulk titanium materials, TiO_2_ NPs and ions are no longer considered bio-inert and ultrafine TiO_2_ is classified as a potential occupational carcinogen by the National Institute for Occupational Safety and Health [1]. Medical implants are a source of endogenous exposure to TiO_2_ NPs and ions, as both mechanical wear and prolonged inflammation can lead to material corrosion [12,42,43,44]. Degradation products from implant corrosion include nano- and macroparticles, free ions, and organometallic compounds [45,46,47]. Inhalation of engineered NPs is one of the most common forms of exogenous occupational exposure to ultrafine TiO_2_ [8,48]. While immune cells, including macrophages, clear TiO_2_ from the alveolar spaces via phagocytosis, overloading caused by excessive particle inhalation limits macrophages’ ability to clear TiO_2_, causing prolonged exposure on the order of years rather than days or months [49].

Previous studies have shown immune responses to titanium-based particles, but few studies have been able to demonstrate immune responses to ionic titanium antigens [37,50,51,52]. This is partially because of the lack of a stable ionic titanium antigen at sufficient concentrations for immunological testing [21,53]. In this study, we were able to stabilize the Ti(IV)-ions in a physiological medium by complexing it to HSA in a 2:1 molar ratio. To our knowledge, this is the first ionic titanium antigen that has successfully induced an immune response in vitro. We propose that the Ti-albumin antigens act as haptens typical for a type IV hypersensitivity reaction, but this must be further investigated in a mechanistic study.

### 3.2. Flow Cytometry

Flow cytometry was used to measure lymphocyte proliferation via reduction of cytoplasmic CFSE concentration (and thereby fluorescence intensity) upon cell division. Most report good correlation of the CFSE-based test with the traditional tritiated thymidine incorporation when analyzing reactivity to common antigens of protein nature [54], and this technique offers additional advantages due to the avoidance of radioactive reagents and the enumeration of proliferative responses from specific lymphocyte subpopulations. The usefulness of CFSE measurements in assessing beryllium sensitivity has been investigated [55,56]. Additionally, immunophenotyping has previously been used to assess immune cell activation in response to metal stimuli, demonstrating that chromium and nickel induce expression of CD25 in CD4+ T cells [57]. Few studies have investigated immunophenotypic changes in response to ionic and NP TiO_2_, though there is evidence that tetravalent ionic titanium induces minor changes in CD25 and a major increase in CD69 expression, and that titanium ions decrease the activation markers CD25 and HLA-DR on dendritic cells [58,59,60]. To our knowledge, combined CFSE proliferation testing and immunophenotyping to analyze metal hypersensitivity has not previously been done. Here we demonstrate that this multiparametric flow cytometry method may be well suited to assess metal sensitivity in patients or employees with exposure to endogenous or exogenous TiO_2_, respectively. Specifically, we identify HLA-DR as a measure of activation after seven days of stimulation and use the CFSE stain to verify proliferative properties of activated cells.

### 3.3. Cytokines

The cytokines that were secreted in response to titanium in this study are primarily produced by monocytes or macrophages, such as IL-10, IL-15, MIP-1β, IFN-γ, and IL-1RA. This is in accordance with publications identifying macrophages as key regulators of both endogenous and exogenous TiO_2_ exposure. According to Gallo et al., the most important cells involved in the inflammatory response to prosthetic particles are the monocyte/macrophage lineage [61]. In addition, histological analysis demonstrates that macrophages aggregate in the lung and contain TiO_2_ after either intratracheal installation or inhalation of nanoscale TiO_2_ in vivo [62,63]. This work further strengthens the argument that both titanium nanoparticles and ionic titanium antigens stimulate an immune response via activation of macrophages/monocytes in patient PBMC cultures. In contrast, there was little or no increase in lymphokine secretion after titanium stimulation, i.e., IL-2, IL-4, IL-5, IL-13, and IL-17 (Figure 3). This agrees with previous studies where neither lymphocyte stimulation nor increased production of lymphokines IL-2 or IL-4 were seen after stimulation with titanium debris from metal implants [52] or by subchronic intravenous exposure to palladium NPs [64].

Other types of metal particles, such as Co-Cr-Mo alloy particles, have been shown to stimulate the inflammasome pathway via secretion of IL-1β and other monocytic cytokines, which suggests that titanium debris could stimulate the same inflammatory pathway [34]. We saw a large increase in IL-1β secretion after stimulation with Ti(IV)-albumin and nano-TiO_2_ (Figure 4a,b). This agrees with previously observed increases in IL-1β after topical hapten application to murine ears or particle stimulation in vitro [35,36,40]. Increased IL-1β secretion has also been seen specifically in response to titanium wear debris from implants and in response to intratracheal installation, inhalation, or intraperitoneal injection of nano-TiO_2_ [8,33,52,65,66,67,68].

Several studies have found that simultaneous increases in TNF-α, GM-CSF, and IL-6 accompany secretion of IL-1β, which we also observed [33,35,36,37,69]. Specifically, TNF-α has been shown to increase 40-fold after macrophage exposure to submicron sized titanium alloy particles for 48 h [37]. The release of these cytokines into sites of metal accumulation in vivo can subsequently contribute to an immune response by recruiting and activating additional immune cells, prolonging immune cell survival, and increasing production of reactive oxygen species (ROS) [70,71]. Our results support previous work, as IL-1β, TNF-α, GM-CSF, and IL-6 were all increased in response to metal stimuli. It has been proposed that IL-1/IL1RN/TNF-α genotyping and cytokine release assay scores may be used as a new tool for individual risk assessment [72], and our data supports the hypothesis that secretion levels may be a good measure of reactivity to nano-TiO_2_ exposure.

### 3.4. Possible Mechanism

Based on the work shown here, innate immunity is critical to initiate the immune response to particulate TiO_2_, as we identify a strong upregulation in pro-inflammatory cytokines classically associated with innate immunity, including TNF-α and IL-1β. As suggested by others, the data support the hypothesis that macrophage activation is induced by TiO_2_ NPs and ions to increase secretion of monocyte and macrophage derived cytokines and promote engulfment of the foreign materials [73].

The pro-inflammatory response observed here could also be in part due to toxic responses, as toxicity studies of TiO_2_ nanoparticles demonstrate signs of apoptosis and necrosis at certain concentrations [74]. Kongseng et al. provided evidence that PBMCs treated with TiO_2_-NPs at concentrations ≥25 μg/mL for 24 h significantly reduced cell viability and significantly increased production of toxic mediators such as ROS and inflammatory cytokines such as IL-6 and TNF-α [75]. However, the viability of the PBMCs in our study was not affected after 24 h and 7 days.

Lastly, it is possible that particulate TiO_2_ exposure induces an adaptive immune response via antigen recognition by memory cells among PBMCs. There is evidence of T-lymphocyte mediated immunity in response to chronic intravenous palladium administration in vivo, as key cytokines involved in adaptive immunity were increased, including interferon gamma (IFNγ), IL-2, and IL-4 [76]. This is in contrast to what was observed after subchronic palladium exposure, which did not induce IFNγ, IL-2, or IL-4 [64]. Our work demonstrates that although lymphocyte proliferation of CD4+ cells was observed at high concentrations of TiO_2_ exposure, IL-2 secretion was not increased. This contradicts the hypothesis that lymphocytes become activated since T-lymphocyte proliferation requires both IL-2 production and IL-2 receptor expression [52,77]. Taken together, these findings suggest that adaptive immunity is activated by particulate metal exposure; however, it is likely subject to temporal regulation and requires chronic exposure, and may also be metal specific.

### 3.5. Limitations

The number of test subjects involved in this study was limited and with a mixed background and thus we did not intend to compare subgroups. Another potential weakness might be the selection of subjects who were referred for skin patch testing due to suspected metal allergy from occupational causes. Six subjects tested positive on nickel. However, these subjects did not seem to respond more to in vitro Ti-exposure, so we did not suspect any cross reactivity to nickel. The Ti substances were not tested on the skin. Future experiments should aim to include two larger well-matched cohorts (with respect to age, clinical history, gender, and exposure) in order to investigate significant differences between relevant test groups.

In future studies, dose–response testing including at least a third stimulation concentration for both may help to identify a toxic threshold. Also, chemical characterization of the antigens must be carried out before use in larger cohort studies (ionic oxidation state, chemical structure, stability, purity, etc.).

## 4. Materials and Methods

### 4.1. Test Subjects

Blood samples were obtained from twenty test subjects; 8 without implants and 12 with titanium-based implants, primarily total joint replacements and metallic stents. The test subjects without implants were recruited through the Department of Occupational Medicine due to suspected contact allergy to nickel. Persons with implants attended regular controls at their respective clinics at Haukeland University Hospital (Table 1).

### 4.2. Ethics

The study was conducted in accordance with the Declaration of Helsinki and was approved by the Western Norway ethics committee (at the Haukeland University Hospital) in Bergen, Norway (ethical permission no. 2013/66/REK Vest) and all test subjects gave informed consent.

### 4.3. Blood Sample Collection and Processing

Blood samples were obtained by venipuncture in sterile BD vacutainer^®^ ACD blood collection tubes. Peripheral blood mononuclear cells (PBMCs) were isolated on the same day as collection in LeucoSep^®^ (Greiner Bio-One, Frickenhausen, Germany) density gradient centrifugation tubes according to the established LeucoSep^®^ method. The PBMC fraction was removed and washed with EMEM medium (Sigma-Aldrich, St. Louis, MO, USA) supplemented with 2.7% *v*/*v* sodium bicarbonate, 1% *v*/*v* l-glutamine, and 1% *v*/*v* HEPES buffer (all from Lonza, BioWhittaker^®^, Basel, Switzerland). PBMCs were suspended in RPMI-1640 medium (HEPES modification with 25 mM HEPES and sodium bicarbonate, Sigma-Aldrich) supplemented with 1% *v*/*v* sodium pyruvate, 2.6% *v*/*v* sodium bicarbonate, 1% *v*/*v* l-glutamine, penicillin streptomycin (97 U/mL pen, 97 U/mL strep), amphotericin B (25 μg/mL) (all from Lonza, BioWhittaker^®^), 2% *v*/*v* d(+) glucose (Sigma-Aldrich, St. Louis, MO, USA) containing either 20% heat-inactivated AB serum or 10% heat-inactivated fetal bovine serum, for cytokine analysis or flow cytometry respectively. Cell concentrations and viability were determined using trypan blue staining and Countess™ automated cell counter (Invitrogen, LifeTechnologies^TM^, Carlsbad, CA, USA).

### 4.4. Titanium Antigens Used for In Vitro Assays

Two forms of titanium antigens were developed, soluble ionic Ti(IV)-complexes and insoluble TiO_2_-nanoparticles, both forms stabilized by albumin.

#### 4.4.1. Ionic Titanium(IV) Albumin Antigens (Ionic-Ti)

Ti(III)citrate was made by mixing TiCl_3_ (0.134 M in HCl) with three-fold molar mass excess of Na_3_citrate [23,78]. The pH was adjusted to pH 3 by NaOH. The solution was stirred for 24 h causing oxidation of the Ti(III)citrate to Ti(IV)citrate [17]. The pH was adjusted to physiological pH 7.4 by NaOH addition and diluted to a concentration of 33.5 mM Ti(IV)citrate.

Human serum albumin (HSA, Sigma-Aldrich, A3782-500MG) (0.2261 g) was dissolved in 3 mL RPMI-1640 medium, followed by the addition of 200 µL Ti(IV)citrate (2:1 Ti:HSA molar ratio). The solutions were allowed to mix overnight to facilitate citrate–albumin exchange. Ti(IV)albumin was separated from residual Ti(IV)citrate in a 10 mL Zeba™ spin desalting column with a cut-off of <1000 Da according to the manufacturer’s protocol. The column was washed with 2 mL RPMI-1640 and centrifuged for an additional 2 min (1000× *g*). The concentration of the final stock solution of 46.0 μg/mL was determined by inductively coupled plasma mass spectrometry (Element 2, Thermo Finnigan, Bremen, Germany). The solution was stored at −20 °C and aliquots were thawed and sterile filtered using the Whatman™ FP20/0.45 CA-S syringe filters. PBMCs were exposed to the ionic Ti(IV)albumin antigen at 0.25 µg/mL and 2.5 µg/mL.

#### 4.4.2. Nano-TiO_2_ Antigens

Commercially pure titanium dioxide (TiO_2_) nanoparticles, 99.9%, consisting of the crystallographic forms anatase and rutile with an average diameter of <100 nm (MW05084, Sigma-Aldrich, St. Louis, MO, USA) were used to make the stock suspension. Physicochemical characterization of the TiO_2_ nanoparticles and preparation of human serum albumin (HSA) stabilized nano-suspensions has previously been shown [28,79]. A 1% TiO_2_ suspension was prepared by dissolving 0.2508 g TiO_2_ in 25 mL of milli-Q water. HSA (Sigma-Aldrich, A3782-500MG) (0.5002 g) was added to a glass bottle and dissolved in RPMI-1640 medium. The HSA solution and the nano-TiO_2_ suspension were stored at −20 ° C and 4 °C, respectively. Before each experiment a 0.1% nano-TiO_2_ suspension (sonicated 1 min at 70%, Sonics VibraCell™) was prepared by combining the HSA solution (500 μL) with the nano-TiO_2_ suspension (100 μL, in RPMI-1640 medium 400 μL). The suspension was sterile filtered immediately (Whatman™ FP20/0.45 CA-S syringe filters) and rotated at 10 rpm until used to minimize agglomeration of particles. The suspension was diluted and PBMCs were exposed to nano-TiO_2_ at 5 µg/mL and 50 µg/mL.

### 4.5. Endotoxin Testing

An endpoint chromogenic LAL test was performed on all test substances and three test subjects samples using the Pierce^®^ LAL Chromogenic Endotoxin Quantitation Kit (ThermoScientific, Pierce Biotechnology, Rockford, IL, USA) according to the manufacturers’ protocol.

### 4.6. Flow Cytometry Analyses

**Figure A1:**
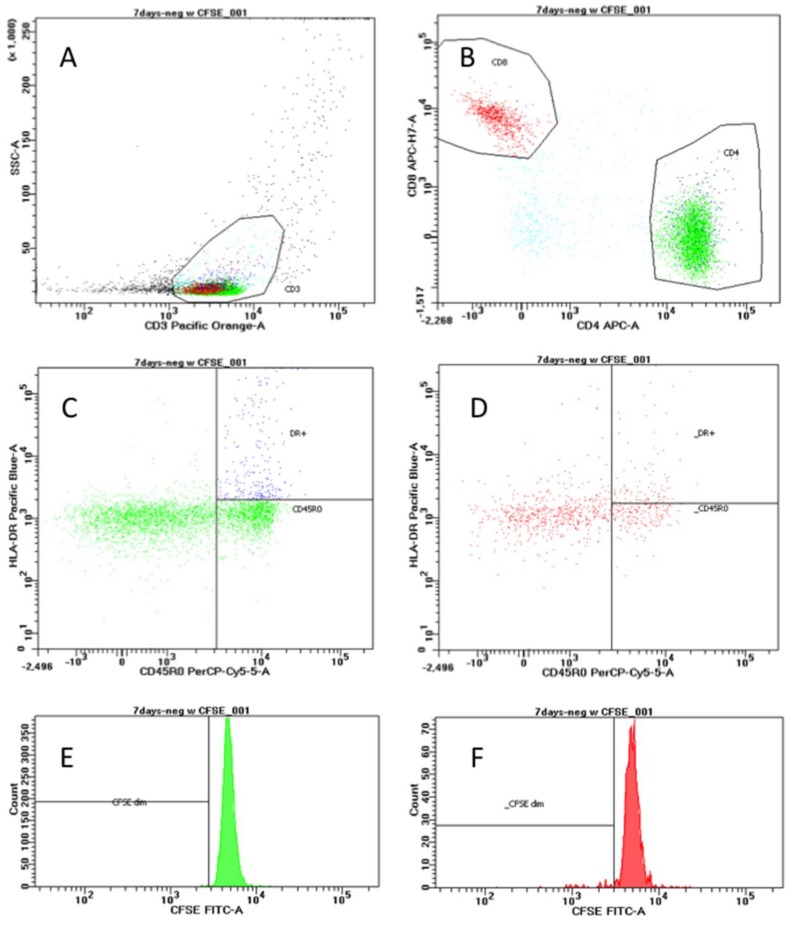
Flow charts showing gating of negative controls. (**A**) Gating on CD3+ lymphoid cells. (**B**) CD4+ cells (green), CD8+ cells red). (**C**) Activated effector T helper cells (CD45RO+/HLA-DR+) in upper right quadrant. (**D**) Activated effector T cytotoxic cells (CD45RO+/HLA-DR+). (**E**) CFSE-staining of CD4+ T cells showing no cell division. (**F**) CFSE-staining of CD8+ T cells showing no cell division.

**Figure A2:**
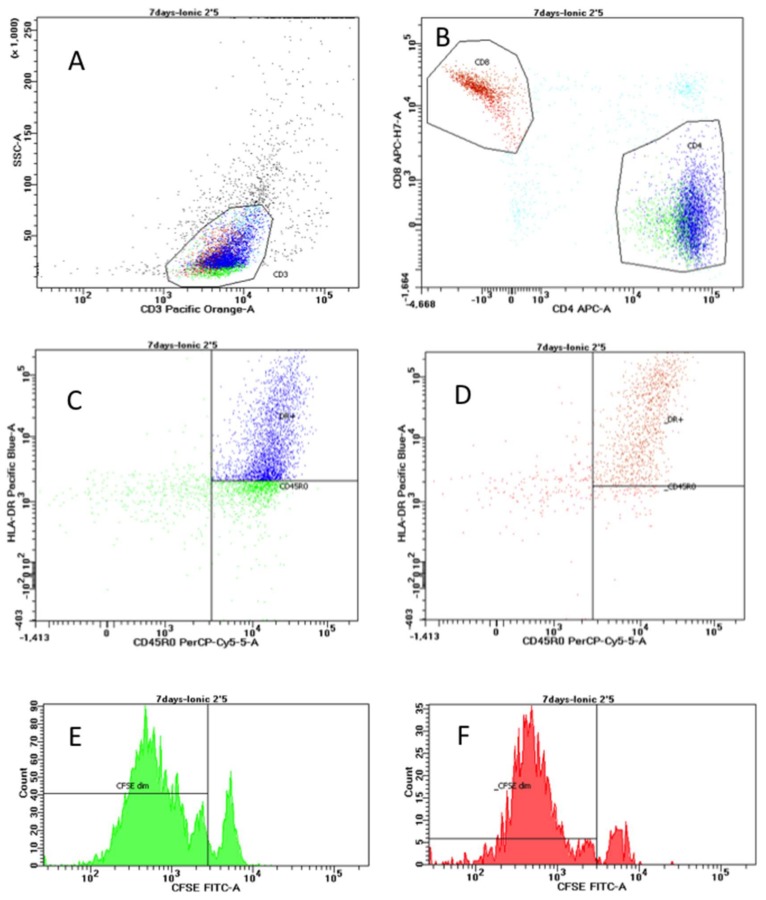
Flow chart example of the gating for the stimulated cell populations (2.5 ug/L ionic-Ti after seven days). (**A**) Gating on CD3+ lymphoid cells. (**B**) CD4+ cells (green), CD8+ cells (red). (**C**) Activated effector T helper cells (CD45RO+/HLA-DR+) in upper right quadrant. (**D**) Activated effector T cytotoxic cells (CD45RO+/HLA-DR+) in upper right quadrant. (**E**) CFSE-staining of CD4+ T cells showing cell division in several generations. (**F**) CFSE-staining of CD8+ T cells showing cell division in several generations.

PBMCs (10 × 10^6^ cells/mL) were cultured in U-bottomed 96-well plates and exposed to metal stimuli at the concentrations specified for each compound above. Phorbol myristate acetate (PMA, Sigma-Aldrich, St. Louis, MO, USA), and Ionomycin (Calbiochem, Merck, Darmstad, Germany) at 10 ng/mL and 1 µg/mL, PHA at 2 µg/mL, and tuberculin purified protein derivative (PPD, Statens Seruminstitutt, Copenhagen, Denmark) at 12.5 µg/mL were used as controls for cell viability and media alone was used as a negative control. In order to enhance antigen stimulation, PBMCs were co-stimulated with FastImmune™ CD28/49d (2 µL/well, except PHA and PMA/Ionomycin) (BD Biosciences, San Jose, CA, USA). Experiments were run in duplicate. PBMC suspensions (total volume 200 µL/well) were incubated at 37 °C and 5% CO_2_ in humidified air for 48 h and seven days. For the seven day analysis, CFSE incorporation was used according to the manufacturer’s protocol (Molecular Probes™, CellTrace™ CFSE Cell Proliferation Kit (C34554)) using 0.25 µL of 5 mM stock CFSE solution per milliliter of cells giving a final working concentration of 1.25 µM. Cells with no CFSE incorporation were used as a negative control. Gating on forward- and side-scatter plots to exclude dead cells CD3+ cells in the lymphoid cluster were analyzed (Appendix A, Figure A1). After 48 h of stimulation, activation-marker expression (CD69) on CD4+ and CD8+, gated from CD3+ T cells was measured using fluorochrome labeled antibodies to CD69 PE, CD4 Pacific Blue, CD3 APC-H7 (BD Biosciences, San Jose, CA, USA), CD8 FITC (Cytognos, Salamanca, Spain) and CD45 Pacific Orange (Invitrogen/Life Technologies, Waltham, MA, USA). After 7 days, cell proliferation and HLA-DR positivity were measured on CD4+ and CD8+ after gating on CD3+ T cells using a different panel of antibodies, in addition to CFSE (Invitrogen/Life Technologies, Waltham, MA, USA) (Appendix A, Figure A2). Included in this panel were antibodies against CD3 Pacific Orange, CD4 APC, CD8 APC-H7 (BD Biosciences, Franklin Lakes, NJ, USA), HLA-DR Pacific Blue (Biolegend, San Diego, CA, USA). All antibodies were titrated for optimal separation and staining intensities of cell populations. Fifty microliters of an immediate premix of titrated antibodies was added to fifty microliters of cells and incubated for 15 min at room temperature in the dark. The cells were analyzed on a BD Canto II flow cytometer (BD Biosciences, Franklin Lakes, NJ, USA), fitted with three lasers to obtain eight fluorochrome parameters in addition to scatter. The proliferation measured via CFSE incorporation was normalized to the negative controls by calculating a stimulation index (SI) as follows: SI = (percentage of stimulated cells CFSE positive)/(mean percentage of unstimulated control cells CFSE positive). SI > 2 was considered significant. Blood samples from 6 of 20 test subjects were included in flow cytometric analysis.

### 4.7. Cytokine Analyses

PBMCs (1.16 × 10^6^ ± 6.4 × 10^5^ cells/mL) were cultured in 24-well plates and exposed to metal stimuli at the concentrations specified for each compound above. PMA/I (10 ng/mL, 1 µg/mL) and PHA (2 µg/mL) were used as controls for cell viability and media alone was used as a negative control. Experiments were run in duplicate. PBMC suspensions (total volume 500 µL/well) were incubated at 37 °C and 5% CO_2_ in humidified air for 24 h. Supernatants were isolated by centrifugation and stored at −20 °C until further analysis and moved to −80 °C for long-term storage. The cytokine levels in PBMC supernatants were obtained using a bead based multiplex cytokine detection kit (LHC0009, Invitrogen/Life technologies, Camarillo, CA, USA) and the assay was carried out according to the manufacturer’s protocol. The multiplex kit measured the following 24 cytokines: IL-1β, IL-10, IFN-α, IL-6, IL-12, Eotaxin, IL-13, IL-15, IL-17, MIP-1α, GM-CSF, MIP-1β, MCP-1, IL-5, IFN-**γ**, TNF-α, IL-1RA, IL-2, IL-7, IP-10, IL-2R, MIG, IL-4, and IL-8 (abbreviations are defined in Table 1). The samples were applied on a Luminex^®^ 100™ instrument (Luminex Corporation, Austin, TX, USA), and data was captured and analyzed by StarStation v.3 software (AppliedCytometry, Dinnington, Sheffield, UK). RANTES was also measured, but data was excluded because most of the concentrations were outside of the measurable range. For all other cytokines, infrequent values outside of the measurable range were assumed to be 0. Blood samples from all twenty test subjects were included in cytokine analysis.

### 4.8. Statistical Analysis

The Wilcoxon matched-pair signed rank test was used to compare CD69 and HLA-DR expression among all conditions (negative controls and low and high concentrations of each metal stimulus). The Wilcoxon matched-pair signed rank test was also used to compare cytokine responses between unstimulated cells with all other conditions (positive controls and Ti antigens). *p*-values < 0.05 were considered to be statistically significant. All tests were performed using the GraphPad Prism statistics software version 7.03.

## 5. Conclusions

We have shown that both nanoparticulate and ionic titanium antigens initiate immune responses in vitro. In general, the higher concentrations of both forms of titanium elicit higher responses than the lower concentrations, and the Ti(IV)-albumin elicits a greater immune response than nano-TiO_2_. This trend was seen in stimulation indices, HLA-DR expression, and in cytokine secretion. Of the 24 cytokines measured, about half of them showed very significant differences after stimulation with both forms of titanium. The majority of cytokines with increased expression were macrophage and monocyte type cytokines, suggesting that immune activation is via this pathway. Our preliminary results show that many of the responses to titanium antigens were general and were induced in all test subjects. Further investigation is also necessary to elucidate the mechanism by which titanium substances induces allergic responses in vivo, and to develop a diagnostic tool for both chronically exposed workers and adverse reactions to implants.

## Figures and Tables

**Table 1:** Test subject information, implant status, and general health

Patient	Sex	Age	Metal Implants	General Health
1	F	1965	-	Healthy. Ni allergic
2	F	1968	-	Healthy. Ni allergic
3	F	1993	-	Healthy. Ni allergic
4	M	1968	-	Healthy. Ni allergic
5	F	1964	-	Healthy. Ni allergic
6	M	1964	-	Healthy. Ni allergic and hand eczema.
7	M	1991	-	Healthy. Hand eczema.
8	M	1973	-	Healthy. Skin allergy. Hand eczema.
9	F	1963	THA ^1^ (1y)	Diabetes, Joint/muscle disease
10	M	1968	THA	Stroke (8 years ago)
11	M	1949	Plates/screws (22 y)	Good
12	M	1943	Bilateral THA (1 y, 3 y)	Good
13	F	1947	Bilateral THA (20 y)	Osteoarthritis. Hip pain.
14	F	1974	Bilateral THA (11 y). Ti heart valve (15 y)	Heart disease, Joint/Muscle disease or rheumatism
15	M	1934	Peripheral stent (2 y). Pacemaker (2 y)	Diabetes, heart disease, Parkinson’s
16	M	1942	Two peripheral stents (12 y, 14 y)	Parkinson’s and heart disease
17	F	1940	Aorta stent, re-stented twice due to restenosis	Heart disease
18	M	1943	Stent graft (10 days)	N/A ^3^
19	F	N/A	Nails/Screws right knee (11 y). TKA ^2^ left (6 y)	High blood pressure, osteoarthritis, rhinitis and eczema
20	F	1984	Dental implants (6 y)	Gastric/ Intestinal disease (IBS) and eczema

^1^ Total Hip Arthroplasty (THA), ^2^ Total Knee Arthroplasty (TKA). ^3^ No information (N/A).
